# Complete genome sequence of *Slackia heliotrinireducens* type strain (RHS 1^T^)

**DOI:** 10.4056/sigs.37633

**Published:** 2009-11-22

**Authors:** Rüdiger Pukall, Alla Lapidus, Matt Nolan, Alex Copeland, Tijana Glavina Del Rio, Susan Lucas, Feng Chen, Hope Tice, Jan-Fang Cheng, Olga Chertkov, David Bruce, Lynne Goodwin, Cheryl Kuske, Thomas Brettin, John C. Detter, Cliff Han, Sam Pitluck, Amrita Pati, Konstantinos Mavrommatis, Natalia Ivanova, Galina Ovchinnikova, Amy Chen, Krishna Palaniappan, Susanne Schneider, Manfred Rohde, Patrick Chain, Patrik D'haeseleer, Markus Göker, James Bristow, Jonathan A. Eisen, Victor Markowitz, Nikos C. Kyrpides, Hans-Peter Klenk, Philip Hugenholtz

**Affiliations:** 1 DSMZ - German Collection of Microorganisms and Cell Cultures GmbH, Braunschweig, Germany; 2 DOE Joint Genome Institute, Walnut Creek, California, USA; 3 Los Alamos National Laboratory, Bioscience Division, Los Alamos, New Mexico, USA; 4 Biological Data Management and Technology Center, Lawrence Berkeley National Laboratory, Berkeley, California, USA; 5 HZI - Helmholtz Centre for Infection Research, Braunschweig, Germany; 6 Lawrence Livermore National Laboratory, Livermore, California, USA; 7 University of California Davis Genome Center, Davis, California, USA

**Keywords:** Gram-positive coccus, anaerobic, asaccharolytic, pyrrolizidine alkaloids, *Coriobacteriaceae*

## Abstract

*Slackia heliotrinireducens* (Lanigan 1983) Wade *et al*. 1999 is of phylogenetic interest because of its location in a genomically yet uncharted section of the family *Coriobacteriaceae,* within the deep branching *Actinobacteria*. Strain RHS 1^T^ was originally isolated from the ruminal flora of a sheep. It is a proteolytic anaerobic coccus, able to reductively cleave pyrrolizidine alkaloids. Here we describe the features of this organism, together with the complete genome sequence, and annotation. This is the first complete genome sequence of the genus *Slackia,* and the 3,165,038 bp long single replicon genome with its 2798 protein-coding and 60 RNA genes is part of the *** G****enomic* *** E****ncyclopedia of* *** B****acteria and* *** A****rchaea * project.

## Introduction

Strain RHS 1^T^ (= DSM 20476 = ATCC 29202 = JCM 14554) is the type strain of the species *Slackia heliotrinireducens* and was originally described by Lanigan in 1976 as *Peptococcus heliotrinreducans* (*sic*) [1] and validly published following an orthographic correction as *Peptococcus heliotrinreducens* in 1983 [2,3]. The strain was later transferred to the genus *Peptostreptococcus* on the basis of its G+C content [4]. 16S rRNA gene sequence analysis indicated that it should not be assigned to the genus *Peptostreptococcus* and therefore the strain was subsequently allocated to the novel genus *Slackia* as *S. heliotrinireducens* [5,6]. The three species of the genus *Slackia*, *S. exigua*, *S. faecicanis*, and *S. heliotrinireducens* form a distinct cluster within the *Coriobacteriaceae*, located in the neighborhood to the genera *Cryptobacterium* and *Collinsella*.

Five additional strains identified as *S. heliotrinireducens* based on their proteolytic enzyme profiles have been isolated from human polymicrobial abscesses [7], but these strains were dissimilar from the type strain as shown by pyrolysis mass spectrometry [8]. With 94% sequence identity (16S rRNA gene), *S. exigua*, the type strain of the closest related species represents the only meaningful (>91%) hit in nucleotide sequence database searches, indicating a complete lack of cultivated and even uncultivated relatives of strain RHS 1^T^ in accessible microbiological diversity. Screening of environmental samples and surveys reported at NCBI BLAST server indicated no closely related phylotypes that can be linked to the species (as of July 2009). Here we present a summary classification and a set of features for *S. heliotrinireducens* RHS 1^T^ Together with the description of the complete genomic sequencing and annotation.

### Classification and features

**Figure 1:**
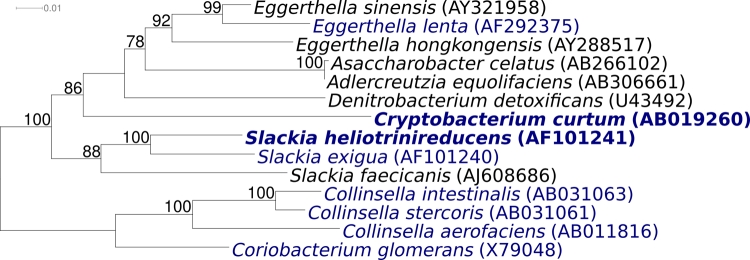
Phylogenetic tree highlighting the position of *S. heliotrinireducens* RHS 1^T^ relative to other type strains within the family *Coriobacteriaceae*. The tree was inferred from 1,422 aligned 16S rRNA characters [9,10] under the maximum likelihood criterion [11], and rooted with type strains of the genera *Collinsella* and *Coriobacterium*. The branches are scaled in terms of the expected number of substitutions per site. Numbers above branches are support values from 1,000 bootstrap replicates, if larger than 60%. Lineages with type strain genome sequencing projects registered in GOLD [12] are shown in blue, published genomes in bold, e.g. the recently published GEBA genomes from *Cryptobacterium curtum* [13], and *Eggerthella lenta* [14].

Figure 1 shows the phylogenetic neighborhood of *S. heliotrinireducens* strain RHS 1^T^ in a 16S rRNA based tree. The sequence of one of the two 16S rRNA genes differs in two nucleotides from the other copy and from the previously published 16S rRNA sequence generated from ATCC 29202 (AF101241).

**Figure 2:**
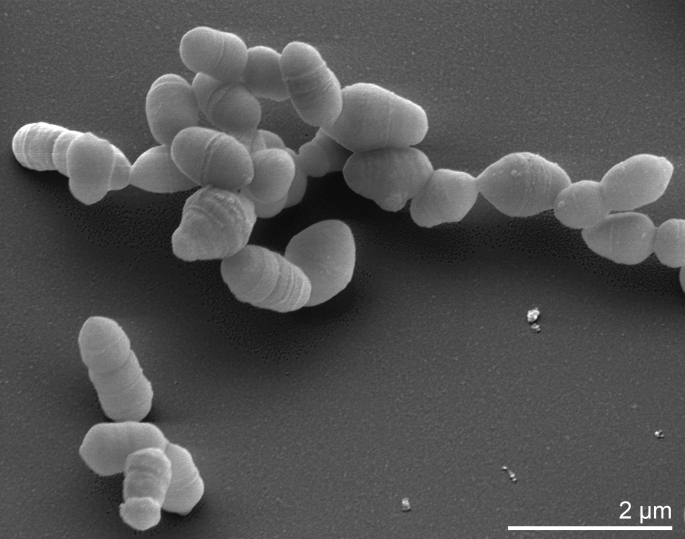
Scanning electron micrograph of *S. heliotrinireducens* RHS 1^T^

*S*. *heliotrinireducens* is Gram-positive, nonmotile, obligatly anaerobic, and does not produce endospores (Table 1). Strain RHS 1 forms cocci or coccobacilli (Figure 2) with a diameter of 0.3 to 0.6 µm and 0.8 x 1.2 µm, respectively [5,6]. The strain grows very slowly on blood agar and forms small translucent, glistening colonies, up to 1 mm in diameter after extensive incubation. It does not utilize carbohydrates, but reduces nitrates and pyrrolizidine alkaloids [5,6]. Reductive cleavage of pyrrolizidines (heliotrine, europine, heleurine, supinine and lasiocarpine) occurs by using hydrogen gas or formate as hydrogen donor [1]. Ammonia is formed from tryptone, yeast extract, adenine, uracil and arginine. Nitrates are completely reduced to ammonia if an appropriate electron donor (H_2_, formate) is present [19]. The strain is bile-sensitive, indole-negative, hydrolyses arginine but not esculin. Does not produce catalase or urease, but is able to dissimilate arginine. Growth is generally stimulated by addition of 0.5% arginine. Metabolic products from *S. heliotrinireducens* grown in prereduced PYG broth are acetic acid, isovaleric acid, and butyric acid in trace amounts [4].

**Table 1:** Classification and general features of *S. heliotrinireducens* RHS 1^T^ in accordance to the MIGS recommendations [15].

**MIGS ID**	**Property**	**Term**	**Evidence code**
	Current classification	Domain *Bacteria*	TAS [16]
		Phylum *Actinobacteria*	TAS [17]
		Class *Actinobacteria*	TAS [18]
		Order *Coriobacteriales*	TAS [18]
		Suborder *Coriobacteridae*	TAS [18]
		Family *Coriobacteriaceae*	TAS [18]
		Genus *Slackia*	TAS [5]
		Species *Slackia heliotrinireducens*	TAS [5]
		Type strain RHS 1	TAS [5]
	Gram stain	positive	TAS [1]
	Cell shape	cocci to coccobacilli	TAS [1]
	Motility	nonmotile	TAS [1]
	Sporulation	nonsporulating	TAS [1]
	Temperature range	mesophile, 30-46°C	TAS [19]
	Optimum temperature	38-42°C	TAS [19]
	Salinity	5g NaCl per l	TAS [5]
MIGS-22	Oxygen requirement	obligate anaerobic	TAS [1]
	Carbon source	asaccharolytic	TAS [1]
	Energy source	arginine, proteolytic	NAS
MIGS-6	Habitat	rumen (sheep)	TAS [1]
MIGS-15	Biotic relationship	free living	NAS
MIGS-14	Pathogenicity	assumed	NAS
	Biosafety level	1 (+)	TAS [20]
	Isolation	rumen of sheep	TAS [1]
MIGS-4	Geographic location	Australia	NAS
MIGS-5	Sample collection time	about 1974	TAS [1]
MIGS-4.1 MIGS-4.2	Latitude – Longitude	not reported	
MIGS-4.3	Depth	not reported	
MIGS-4.4	Altitude	not reported	

Evidence codes - IDA: Inferred from Direct Assay (first time in publication); TAS: Traceable Author Statement (i.e., a direct report exists in the literature); NAS: Non-traceable Author Statement (i.e., not directly observed for the living, isolated sample, but based on a generally accepted property for the species, or anecdotal evidence). These evidence codes are from the Gene Ontology project [21]. If the evidence code is IDA, then the property should have been directly observed for a living isolate by one of the authors, or an expert mentioned in the acknowledgements.

Almost nothing is known about the chemotaxonomy of strain RHS 1^T^, except that its predominant cellular fatty acid is C18:1 [4].

## Genome sequencing information

### Genome project history

This organism was selected for sequencing on the basis of its phylogenetic position, and is part of the *** G****enomic* *** E****ncyclopedia of* *** B****acteria and* *** A****rchaea * project. The genome project is deposited in the Genome OnLine Database [12] and the complete genome sequence is in GenBank. Sequencing, finishing and annotation were performed by the DOE Joint Genome Institute (JGI). A summary of the project information is shown in Table 2.

**Table 2:** Genome sequencing project information

**MIGS ID**	**Property**	**Term**
MIGS-31	Finishing quality	Finished
MIGS-28	Libraries used	Three genomic libraries: two Sanger-8kb pMCL200 and fosmid pcc1Fos Sanger libraries and one 454 pyrosequence standard library
MIGS-29	Sequencing platforms	ABI3730, 454 GS FLX
MIGS-31.2	Sequencing coverage	6x Sanger; 20× pyrosequence
MIGS-30	Assemblers	Newbler version 1.1.02.15, phrap
MIGS-32	Gene calling method	Genemark 4.6b, GenePRIMP, tRNAScan-SE-1.23, infernal 0.81
	INSDC ID	CP001684
	Genbank Date of Release	August 28, 2009
	GOLD ID	Gc01094
	NCBI project ID	20831
	Database: IMG-GEBA	2500901757
MIGS-13	Source material identifier	DSM 20476
	Project relevance	Tree of Life, GEBA

### Growth conditions and DNA isolation

*S. heliotrinireducens* strain RHS 1^T^, DSM 20476, was grown anaerobically in DSMZ medium 104 (PYG) [22]; at 37°C. DNA was isolated from 1-1.5 g of cell paste using Qiagen Genomic 500 DNA Kit (Qiagen, Hilden, Germany) with a modified protocol for cell lysis (FT), as described in Wu *et al.* [23].

### Genome sequencing and assembly

The genome was sequenced using a combination of Sanger and 454 sequencing platforms. All general aspects of library construction and sequencing performed at the JGI can be found on the JGI website (http://www.jgi.doe.gov/). 454 Pyrosequencing reads were assembled using the Newbler assembler version 1.1.02.15 (Roche). Large Newbler contigs were broken into 3,507 overlapping fragments of 1,000 bp and entered into the assembly as pseudo-reads. The sequences were assigned quality scores based on Newbler consensus q-scores with modifications to account for overlap redundancy and to adjust inflated q-scores. A hybrid 454/Sanger assembly was made using the parallel phrap assembler (High Performance Software, LLC). Possible mis-assemblies were corrected with Dupfinisher or transposon bombing of bridging clones [24]. Gaps between contigs were closed by editing in Consed, custom primer walk or PCR amplification. A total of 1,433 Sanger finishing reads were produced to close gaps, to resolve repetitive regions, and to raise the quality of the finished sequence. The final assembly consists of 21.045 Sanger and 205,234 pyrosequence (454) reads. Together all sequence types provided 26× coverage of the genome. The error rate of the completed genome sequence is less than 1 in 100,000.

### Genome annotation

Genes were identified using GeneMark [25] as part of the genome annotation pipeline in the Integrated Microbial Genomes Expert Review system (http://img.jgi.doe.ogv/er) [26], followed by a round of manual curation using the JGI GenePRIMP pipeline (http://geneprimp.jgi-psf.org/) [27]. The predicted CDSs were translated and used to search the National Center for Biotechnology Information (NCBI) nonredundant database, UniProt, TIGRFam, Pfam, PRIAM, KEGG, COG, and InterPro databases. The tRNAScanSE tool [28] was used to find tRNA genes, whereas ribosomal RNAs were found by using the tool RNAmmer [29]. Other non coding RNAs were identified by searching the genome for the Rfam profiles using INFERNAL (v0.81) [30]. Additional gene prediction analysis and manual functional annotation was performed within the Integrated Microbial Genomes (http://img.jgi.doe.gov/) platform [31].

### Metabolic network analysis

The metabolic Pathway/Genome Database (PGDB) was computationally generated using Pathway Tools software version 12.5 [32] and MetaCyc version 12.5 [33], based on annotated EC numbers and a customized enzyme name mapping file. It has undergone no subsequent manual curation and may contain errors, similar to a Tier 3 BioCyc PGDB [34].

## Genome properties

**Figure 3:**
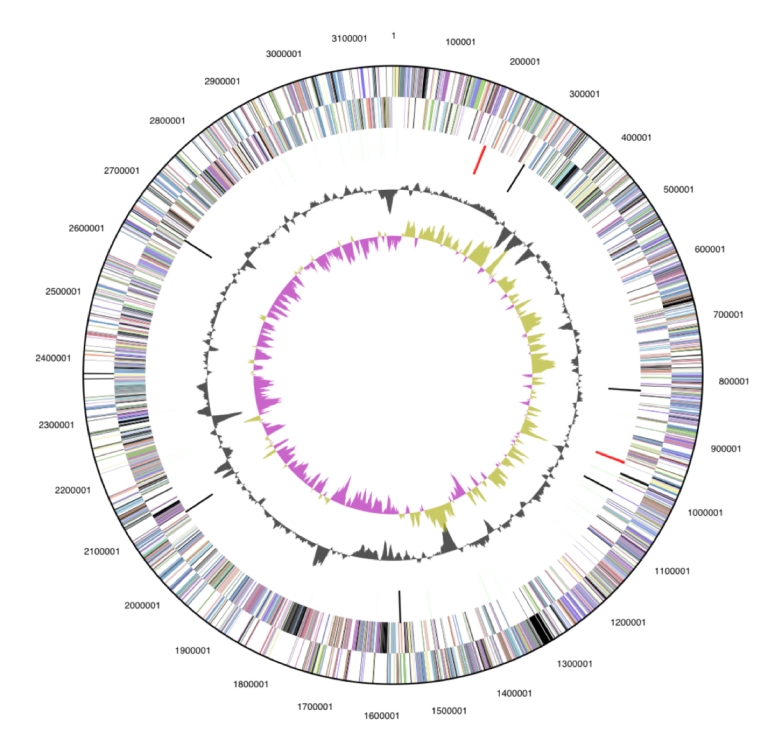
Graphical circular map of the genome. From outside to the center: Genes on forward strand (color by COG categories), Genes on reverse strand (color by COG categories), RNA genes (tRNAs green, rRNAs red, other RNAs black), GC content, GC skew.

**Figure 4:**
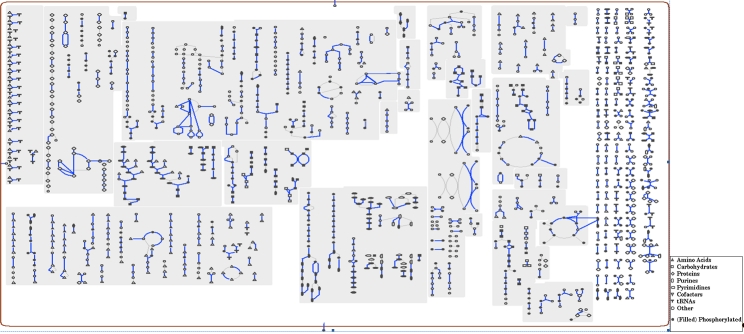
Schematic cellular overview diagram of all pathways of the *S. heliotrinireducens* RHS 1^T^ metabolism. Nodes represent metabolites, with shape indicating class of metabolite (see key to right). Lines represent reactions.

The genome is 3,165,038 bp long and comprises one main circular chromosome with a 60.2% GC content (Table 3 and Figure 3). Of the 2,858 genes predicted, 2,798 were protein coding genes, and 60 RNAs; 33 pseudogenes were also identified. The majority of the protein-coding genes (70.6%) were assigned with a putative function, while those remaining were annotated as hypothetical proteins. The properties and the statistics of the genome are summarized in Table 3. The distribution of genes into COGs functional categories is presented in Table 4, and a cellular overview diagram is presented in Figure 4, followed by a summary of metabolic network statistics shown in Table 5.

**Table 3:** Genome Statistics

**Attribute**	**Value**	**% of Total**
Genome size (bp)	3,165,038	100.00%
DNA coding region (bp)	2,756,714	87.10%
DNA G+C content (bp)	1,905,720	60.21%
Number of replicons	1	
Extrachromosomal elements	0	
Total genes	2,858	100.00%
RNA genes	60	2.03%
rRNA operons	2	
Protein-coding genes	2,798	97.90%
Pseudo genes	33	1.15%
Genes with function prediction	2,014	70.52%
Genes in paralog clusters	433	15.15%
Genes assigned to COGs	1,969	68.94%
Genes assigned Pfam domains	1,977	69.22%
Genes with signal peptides	562	19.66%
Genes with transmembrane helices	123	4.30%
CRISPR repeats	0	

**Table 4:** Number of genes associated with the general COG functional categories

**Code**	**Value**	**% age**	**Description**
J	139	5.0	Translation, ribosomal structure and biogenesis
A	0	0.0	RNA processing and modification
K	208	7.4	Transcription
L	134	4.8	Replication, recombination and repair
B	1	0.0	Chromatin structure and dynamics
D	25	0.9	Cell cycle control, mitosis and meiosis
Y	0	0.0	Nuclear structure
V	48	1.7	Defense mechanisms
T	107	3.8	Signal transduction mechanisms
M	93	3.3	Cell wall/membrane biogenesis
N	3	0.1	Cell motility
Z	0	0.0	Cytoskeleton
W	0	0.0	Extracellular structures
U	30	1.1	Intracellular trafficking and secretion
O	83	3.0	Posttranslational modification, protein turnover, chaperones
C	229	8.2	Energy production and conversion
G	68	2.4	Carbohydrate transport and metabolism
E	151	5.4	Amino acid transport and metabolism
F	58	2.1	Nucleotide transport and metabolism
H	109	3.9	Coenzyme transport and metabolism
I	66	2.4	Lipid transport and metabolism
P	101	3.6	Inorganic ion transport and metabolism
Q	33	1.2	Secondary metabolites biosynthesis, transport and catabolism
R	319	11.4	General function prediction only
S	155	5.5	Function unknown
-	829	29.6	Not in COGs

**Table 5:** Metabolic Network Statistics

**Attribute**	**Value**
Total genes	2,856
Enzymes	457
Enzymatic reactions	750
Metabolic pathways	156
Metabolites	576
